# Supplementary material for: Longitudinal association between e-cigarette use and respiratory symptoms among US adults: Findings from the Population Assessment of Tobacco and Health Study Waves 4–5
Source: PLoS One. 2024 Feb 29;19(2):e0299834. doi: 10.1371/journal.pone.0299834 (PMC10903800; doi:10.1371/journal.pone.0299834)
Supplement: S1 Table — (DOCX) [file pone.0299834.s001.docx]

**S1 Table. Number of days using e-cigarettes and smoking combustible cigarettes in the past 30 days at Wave 4**

|  | n | (%^a^) |
| --- | --- | --- |
| Among current e-cigarette users (n = 1,628) | | |
| 1 – 9 days | 989 | (56.0) |
| 10 – 19 days | 124 | (7.1) |
| 20 – 29 days | 72 | (5.3) |
| 30 days | 434 | (31.6) |
| Unreported | 9 | (0.1) |
| Among current cigarette smokers (n = 5,266) | | |
| 1 – 9 days | 1991 | (37.1) |
| 10 – 19 days | 300 | (5.7) |
| 20 – 29 days | 240 | (4.6) |
| 30 days | 2735 | (52.6) |
| *Note.*  ^a^Weighted proportion |  |  |
