# Supplementary material for: Longitudinal association between e-cigarette use and respiratory symptoms among US adults: Findings from the Population Assessment of Tobacco and Health Study Waves 4–5
Source: PLoS One. 2024 Feb 29;19(2):e0299834. doi: 10.1371/journal.pone.0299834 (PMC10903800; doi:10.1371/journal.pone.0299834)
Supplement: Attachment — Submitted filename: EC Use and Respiratory Health Response to Reviewers.docx [file pone.0299834.s002.docx]

**Reviewer #1**

**COMMENT 1:** This article is an interesting examination of the respiratory symptoms experience by dual users of e-cigarettes and combustible tobacco products using wave 4 and 5 of the PATH study. While I think this article is relevant and is an important addition to the literature, I do think a major revision is necessary to clarify the product user groups, study methods, and analyses.

**RESPONSE 1:** We appreciate the positive comments, and have made revisions accordingly. Please see details below.

**COMMENT 2:** Combustible tobacco use and more specifically, combustible cigarettes pose serious risks to human health. While e-cigarettes are not without harm, there is well documented support that e-cigarette aerosol contains fewer numbers and lower levels of toxicants than smoke from combustible cigarettes. Incorporating or at least reporting the past 30-day frequencies and product use category frequencies (especially by combustible product category), is needed.

**RESPONSE 2:** We have created a supplemental table, reporting numbers of days of e-cigarette use and cigarette smoking in the past 30 days at Wave 4.

**COMMENT 3:** Another factor that is likely important in understanding the respiratory risk of dual current combustible tobacco use and e-cigarette use that missing is the number of combustible products used in conjunction with e-cigarette use status. I understand all of these variables cannot always be included in models. However, I would at least consider reporting frequency of use as a table, and a note in the limitation section (e.g. unable to examine the frequency of past 30-day use or incorporate the number of combustible products used in the analyses due to sample size).

**RESPONSE 3:** As described in RESPONSE 2, a supplemental table containing the frequency of e-cigarette use and cigarette smoking has been created. We also revised the Limitation section and added a few sentences: “Second, current e-cigarette users included occasional users and regular users. Similarly, current combustible tobacco smokers included light smokers and heavy smokers, as well as individuals who smoked single combustible tobacco product and those who smoked multiple tobacco products. Future studies are needed to investigate whether these tobacco use patterns have any impact on respiratory health conditions.”

**COMMENT 4:** If the article is examining the risk of dual use of e-cigarettes with combustible products, a bit of restructuring would help improve the clarity of this message to the reader.

**RESPONSE 4:** We wanted to clarify that this study assessed the effect of e-cigarette use on respiratory symptoms. We did the analyses in 3 subgroups, including A) never combustible tobacco smokers, B) former combustible tobacco smokers, and C) current combustible tobacco smokers. The Results and Discussion are structured by combustible tobacco smoking status.

**COMMENT 5:** Title: I think the authors should consider adjusting the title to clarify that this is the examination of e-cigarettes and combustible tobacco dual use(?) or at least have combustible tobacco in the title.

**RESPONSE 5:** We could modify the title as “Longitudinal association between e-cigarette use and respiratory symptoms among US adults by combustible tobacco smoking status: Findings from the Population Assessment of Tobacco and Health Study Waves 4 – 5”. But the new title may be too long. We defer to the editor to suggest if it is necessary revise the title.

**COMMENT 6:** Abstract: I think in line 34, if “combustible tobacco smokers” was placed after former (to read as: “Among current and former combustible tobacco smokers and current e-cigarette..” instead of “Among current combustible tobacco smokers, former, and current e-cigarette..” Or maybe I am confused at the specific user groups referenced here? Please clarify in the methods the specific user groups to better understand the relationships examined.

**RESPONSE 6:** As explained in RESPONSE 4, this paper including Abstract is structured by combustible tobacco smoking status. We have clarified in Background that “We assessed longitudinal effects of e-cigarette use on respiratory symptoms in a nationally representative sample of US adults by combustible tobacco smoking status.”

We have also revised the Methods of Abstract to clarify the reference group: “Weighted lagged logistic regression models were performed to examine the association between e-cigarette use status at Wave 4 (former/current vs. never use) and important respiratory symptoms at Wave 5 by combustible tobacco smoking status (i.e., never/former/current smokers), adjusting for…”

**COMMENT 7:** Abstract: I would also clarify in the results section what your reference group is (i.e., never users? Current dual users vs. exclusive e-cigarette users?) when discussing the findings.

**RESPONSE 7:** We have added this information in the Methods of Abstract.

**Introduction:
COMMENT 8:** Citation 14 found that dual use of e-cigarettes and cigarettes was associated with respiratory symptoms compared to those who used e-cigarettes exclusively or were a non-current user of either product. Please review for clarity/accuracy.

**RESPONSE 8:** We agree that Citation 14 study includes a comparison between dual use vs. exclusive e-cigarette use. Their findings based on this comparison are about impacts of cigarettes on respiratory health, which is beyond the scope of our study. Our study examined the effects of e-cigarette use on respiratory health. We only summarized findings from published studies on the association between e-cigarette use and respiratory health.

**COMMENT 9:** The sentences following the reference to citation 16 (lines 70-71, i.e., “Such association is observed among never combustible cigarette smokers but not among current combustible cigarette smokers.”) and the following sentence (lines 71-72, i.e., “The results suggested that e-cigarettes pose respiratory risks, but add no additional risk independent of that from cigarette smoking”) are unclear. Please clarify what is mean by such association and review reference 16 for accuracy. I believe the authors only controlled for combustible use statistically and did not make specific comparisons to those who were former or current combustible tobacco users, as such I am not sure "but add no additional risk independent of that from cigarette smoking" means in this context.

**RESPONSE 9:** We have deleted those sentences. We summarized the findings of Citation 16 as “…e-cigarette use (former/current vs. never use) was associated with increased odds of incident respiratory symptoms, adjusting for combustible tobacco smoking [16]”.

**COMMENT 10:** I would suggest that the authors give some context to what the respiratory symptom index is, what are “functionally important respiratory symptoms,” and what an increase in the reference index means (e.g., are higher scores related to more symptoms? Worse respiratory function?).

**RESPONSE 10:** We have added more details about the respiratory symptom index. In the Introduction section, we added the following information.

*Respiratory symptom index is derived based on seven PATH items that assess wheezing and cough [17]. Both symptoms are indicative of airway obstruction. Respiratory symptom index ranges from 0 to 9, with higher index value indicating more severe symptoms. A cutoff value of ≥2 has been validated to indicate important respiratory symptoms for PATH adult samples [17].*

In the Key Measures section, we added the following information.

*Following prior research [15-18], we calculated the respiratory symptom index based on six wheezing items and a nighttime dry cough item (Table 1).* *With a range of 0 – 9, higher index values indicate more respiratory symptoms. When used as the outcome variable, an index score of ≥2 was defined as having important respiratory symptoms [17].*

We also created Table 1 to display the seven wheezing/cough items and how the index was calculated based on the seven measures.

**COMMENT 11:** Further, the authors write that current e-cigarette use was associated with worsening respiratory symptoms, but in the article referenced (article #15), those authors found that there was largely no increased symptom risk for exclusive use of several products including e-cigarettes (after adjustment for pack-years and marijuana) and that the risk ratio for several products including e-cigarettes was significantly lower compared to exclusive use of cigarettes. Maybe the context is missing for the risk associated with worsening respiratory symptoms? Please review and/or clarify.

**RESPONSE 11:** The reference #15 reported that exclusive current e-cigarette use (vs. non-current e-cigarette use) was associated with respiratory symptom index changing from <2 (at Wave 2) to ≥2 (at Wave 3) on page 1614. We believe that we have appropriately summarized the results from Citation #15.

**Methods:
COMMENT 12:** In the Study Population section, it would be helpful to clarify if e-cigarette users needed to be a dual combustible product user to be included in your final sample. It is unclear currently how the inclusion/exclusion criteria are listed if exclusive e-cigarette users were excluded from the analytic sample.

**RESPONSE 12:** We have created Figure 1 to display the final sample. Briefly, study sample include respondents who met following criteria: 1) aged 18 years or older; 2) completed both Wave 4 and Wave 5 surveys; 3) self-report no history of respiratory diseases at Wave 4; and 4) had no missing data on analytic variables in both Waves.

**COMMENT 13:** For the respiratory symptom index, I would review the references cited to make sure you are accurately reflecting those who used/categorized respiratory outcomes similarly or different. For example, citation 15 seems to have used this index and maybe the exact same way as suggested from introduction, but I am not sure. Please review citations for accuracy.

**RESPONSE 13:** We agree that reference #15 used the same method to create the respiratory symptom index and therefore, cited it in the relevant texts.

**COMMENT 14:** I think it would be helpful to readers to present a bit more information about how the symptom index is calculated (e.g., are the yes/no responses counted toward the value of 9? Is this just an average of the frequency of episodes across all variables?). Maybe that would provide more clarity on what the cut-off value is indicative of (2 more symptoms in the past 12 months?). Additionally, I would clarify the dichotomization of the index (from a continuous score) for the use in the logistic regression analyses.

**RESPONSE 14:** We have created Table 1 to show the seven items for calculating the respiratory symptom index, answer options and points for each item.

**COMMENT 15:** In the e-cigarette and combustible smoking section, I would suggest some changes in how the user groups are labeled and a bit more clarity in how each group was created. I think “Ever Use” is a more accurate category, based on the limited information presented rather than “former use.” For example, from a questionnaire item like “Have you ever used an e-cigarette product with nicotine?” (yes/no) you can’t assume that the individual became a frequent user of that product and quit (like the term “former” suggests). Similarly, in the cigarette literature, a former smoker is typically defined as having smoked more than 100 cigarettes in their lifetime. I don’t think having ever smoked (even a puff) of a cigarette equates to being categorized as a former smoker. I would also encourage the authors to consider using a cut off for the number of days in the past 30-day used to define current use (see for example for using 5 or greater days in the past-30 days: Amato, M. S., Boyle, R. G., & Levy, D. (2016). How to define e-cigarette prevalence? Finding clues in the use frequency distribution. Tobacco control, 25(e1), e24-e29.).

**RESPONSE 15:** We agree that some terms related to e-cigarette use (including how to define current use) may be controversial in the literature. We followed the PATH Study Codebook to define never/former/current e-cigarette use in this study. We added a sentence in the Limitation that “…current combustible tobacco smokers included light smokers and heavy smokers, as well as individuals who smoked single combustible tobacco product and those who smoked multiple tobacco products.…”

**COMMENT 16:** I might consider using “dual users of e-cigarettes and combustible tobacco products” to help clarify the user groups to the reader.

**RESPONSE 16:** We used the term “dual use” when discussing implications and in Conclusions section.

**Results:
COMMENT 17:** I think a table is needed which outlines the frequencies of and past 30-day (number of days) product use by study wave. It would be helpful to understand the quantity and frequency of use as it relates to the respiratory index.

**RESPONSE 17:** We have added a supplemental table, reporting e-cigarette use and cigarette smoking frequencies in past 30-day. We don’t have enough power to examine the effect of e-cigarette use frequency on respiratory symptom stratified by combustible tobacco smoking status. The cell size of current e-cigarette use and never combustible tobacco smoking is 65. If dividing current e-cigarette use by its frequency, the cell size will be too small.

**COMMENT 18:** In general, I would clarify when the results are referencing the entire sample (including never users) and when the results reference each product use category. For example, on page 16, line 162, and lines 168-169 is this among all participants?

**RESPONSE 18:** We have made revisions accordingly. When referring to the entire sample, we specified “the sample ….” When referring to a subgroup (e.g., never combustible tobacco smokers), we specified the subgroup.

**COMMENT 19:** The results are hard to interpret without understanding the user groups more clearly. For example, lines 163-166 and 166-168 indicate significant differences in the respiratory status by former and current combustible tobacco use status, but do these results include dual users of e-cigarettes? Or are they combustible product users only?

**RESPONSE 19:** As explained in RESPONSE 18, we have revised the Results and hope it is easier to read compared to the last version.

**COMMENT 20:** I think a key piece missing from the results are never e-cigarette users x combustible tobacco use status analyses. I believe analyses only examine using e-cigarettes x combustible tobacco use status (never, “former,” and current) but I did not see any analyses examining combustible tobacco use status x never e-cigarette use and how that compare to the outcomes presented currently. I think it is important to understand the respiratory risk comparisons between that user group (never e-cigarette vs. ever, and current combustible use) and the e-cigarette “dual” user groups (e-cigarette ever, current vs. ever, current combustible).

**RESPONSE 20:** This study investigated the effect of e-cigarette use on respiratory symptoms. Never e-cigarette use represented the reference group in our logistic regression analyses. The suggested analyses are more about the impact of combustible tobacco on respiratory health, which is beyond the scope of this study.

**Discussion:
COMMENT 21:** Overall, thinking about a potential harm reduction strategy that includes e-cigarettes, I think that saying e-cigarettes present a risk to developing independent of combustible tobacco is relevant (and important!). However, I think the relativity of this risk (of e-cigarette exclusive use compared to combustible tobacco/cigarettes compared to dual use of both types of products) is a key piece missing to balance the discussion. This might help clarify that the message (I think!) about the unique respiratory risk associated with dual use patterns of combustible tobacco and e-cigarette use.

**RESPONSE 21:** We agree the risk of e-cigarettes is small compared to that from combustible tobacco or dual tobacco use. Relative risk of e-cigarettes versus combustible tobacco is beyond the scope of our analyses. We did not seek to compare e-cigarettes with combustible tobacco in this study.

**Reviewer #2**

**COMMENT 1:** This Ms uses data from two waves of the PATH study to determine the relation between use of e-cigarettes and (apparently) onset of wheezing symptoms. Strengths of the study are in data from a representative sample, tests of longitudinal effects, and ability to control for several demographic covariates and (in some way) for prior respiratory symptoms. The authors studied both current and former e-cigarette use and conducted stratified analyses subgrouped on combustible cigarette status, finding significant effects among both current and former smokers. The results may have implications for informing current discussions about the health risks of e-cigarette use. I found strengths in the study but there were several issues that seemed unclear.

**RESPONSE 1:** We appreciate the positive comments, and have revised the paper as requested. Please see details below.

**COMMENT 2:** The introduction around line 54 says that previous results were inconsistent. But when I looked at these studies and ones subsequently discussed I found that they all showed significant associations of at least one e-cigarette index with some types of respiratory symptoms. This doesn’t seem inconsistent to me.

**RESPONSE 2:** We revised the statement which now reads “*Longitudinal research surrounding the respiratory risk of e-cigarette use is limited [12-16]*”.

Inconsistent results have been described in Paragraph 3 of Introduction:

*Three longitudinal studies used self-reported respiratory symptoms as the outcome and found contradictory results [14-16]. Reddy et al.[14] analyzed Waves 3 – 4 PATH data and found that, among respondents with no wheezing or dry cough at Wave 3,* *e-cigarette use (current vs. non-current use) at Wave 3 was associated with increased odds of reporting wheezing or dry cough at Wave 4 among current tobacco smokers but not among non-current tobacco users. In contrast, Sargent et al.[15] analyzed Waves 2 – 3 PATH data and used a novel, validated index of functionally important respiratory symptoms (hereafter referred to as “respiratory symptom index”) as the outcome. The study found that, among adults with no respiratory disease at Wave 2,* *e-cigarette use (current vs. non-current use) at Wave 2 was associated with worsening of respiratory health at Wave 3 (i.e., respiratory symptom index changing from <2 to ≥2 in Wave 2 through Wave 3) among non-cigarette smokers but not among current cigarette smokers [15].*

**COMMENT 3:** I had difficulty understanding the criterion variable. First, the criterion variable is described in the abstract and text as “important respiratory symptoms.” However, the measure employed is essentially an index of wheezing when in fact there are many other respiratory symptoms reflecting important health conditions (e.g., asthma, bronchitis) so I thought the language was misleading. Further it is sometimes said that the index was based on “core wheezing symptoms” but there was no discussion of what non-core wheezing symptoms might be.

**RESPONSE 3:** We have added more details about the respiratory symptom index. In the Introduction section, we added the following information.

*Respiratory symptom index is derived based on seven PATH items that assess wheezing and cough [17]. Both symptoms are indicative of airway obstruction. Respiratory symptom index ranges from 0 to 9, with higher index value indicating more severe symptoms. A cutoff value of ≥2 has been validated to indicate important respiratory symptoms for PATH adult samples [17].*

In the Key Measures section, we added the following information.

*Following prior research [15-18], we calculated the respiratory symptom index based on six wheezing items and a nighttime dry cough item (Table 1). With a range of 0 – 9, higher index values indicate more respiratory symptoms. When used as the outcome variable, an index score of ≥2 was defined as having important respiratory symptoms [17].*

In Statistical Analysis section, we explained that:

*Separate lagged logistic regression was performed to examine the association between Wave 4 e-cigarette use status (former/current vs. never) and important respiratory symptoms at Wave 5 by combustible tobacco smoking status, adjusting for Wave 4 respiratory symptom index (as a continuous variable) and covariates…*

We agree there are myriad respiratory symptoms not captured in the respiratory symptom index.  **COMMENT 4:** The second point of ambiguity for me was whether the criterion measure was binary or continuous. The method section says that reports of symptoms were obtained with nine items and these were combined in an index. Ok, But at other points the continuous score was apparently dichotomized (>2 symptoms vs. <2 symptoms) and the continuous information discarded. Why? And where did this cutpoint come from. Without any justification it seems arbitrary. In the Results section, material at the top of page 10 seems to be dealing with a continuous criterion variable, but the material at page 10 bottom seems to be describing an incidence analysis with a binary criterion variable.

**RESPONSE 4:** As explained in RESPOSNE 3, we have added a sentence in Key Measures to clarify when the index was used as a binary variable: “*With a range of 0 – 9, higher index values indicate more respiratory symptoms. When used as the outcome variable, an index score of ≥2 was defined as having important respiratory symptoms [17].*” We also clarified in Statistical Analysis that “*Separate lagged logistic regression was performed to examine the association between Wave 4 e-cigarette use status (former/current vs. never) and important respiratory symptoms at Wave 5 by combustible tobacco smoking status, adjusting for Wave 4 respiratory symptom index (as a continuous variable) and covariates…*”

**COMMENT 5:** The third ambiguity is how the analyses were conducted. It is said that the analytic sample was persons who had no respiratory disease at baseline and that logistic regression analyses tested for prediction of a binary criterion variable at follow-up. Ok. But at other points it is said that the analyses controlled for prior respiratory symptoms, suggesting a continuous analysis. And Table 4 is mysterious because it does not say whether the criterion variable was dichotomous or continuous, and what appears to be a continuous covariate (“Respiratory symptom index”) is listed as one of the variables in the model, so I was unclear on what happened. Table 4 implies but does not clarify whether all the predictors were entered simultaneously. Thus I ended up being uncertain what was actually done and whether this was a true incidence analysis or some variant.

**RESPONSE 5:** People with no diagnosis of respiratory disease(s) may have shown respiratory symptom(s) such as whizzing and dry cough. Using respiratory symptom index as the outcome allows us to capture early respiratory symptoms indicative of airway obstruction.

In our analyses, among adults with no history of respiratory disease at Wave 4, we tested the association between Wave 4 e-cigarette use status and important respiratory symptoms at Wave 5. We controlled for Wave 4 respiratory symptom index because more severe respiratory symptoms (reflected by greater index scores) at Wave 4 predict important respiratory symptoms at Wave 5.

**COMMENT 6:** The discussion was respectable but left out the fact that a number of studies have found significant associations of e-cigarette use with respiratory disease among nonsmokers. This does not negate the conclusions of the present Ms but I think more context could be helpful.

**RESPONSE 6:** We have a paragraph that discussed results observed among never combustible tobacco smokers. We explained that two previous studies using PATH data found positive association between e-cigarette use and respiratory symptoms among nonsmokers.

**COMMENT 7:** In summary, I think this was basically a sound study and there are replicated results with health implications in there somewhere. But I found the presentation confusing at points and was left uncertain about whether this was an incidence analysis or some kind of onset analysis that controlled for baseline symptom levels.

**RESPONSE 7:** We have revised the Methods section and hope it is now easier to read compared to the last version. In brief, we examined the association between baseline e-cigarette use status and respiratory symptoms at follow-up with adjustment for baseline respiratory symptom index. The analysis was conducted among adults with no history of respiratory diseases at baseline.

**Specific comments:
COMMENT 8:** The abstract is variable on reporting accuracy. Some portions are correct but the summary leaves out the fact that elevated risks of e-cigarette use were found among former smokers as well as among current smokers.

**RESPONSE 8:** In the Results of Abstract, we reported the association between e-cigarette use and respiratory symptoms for each type of combustible tobacco smokers. In the Conclusion of Abstract, we emphasized the result observed among current combustible tobacco smokers. We did not discuss former combustible tobacco smokers because first, more studies are needed considering the complex scenarios of former combustible tobacco smoking combined with former/current e-cigarette use. Second, we cannot add more discussions due to word limitation.

**COMMENT 9:** line 50: Propylene glycol is a toxicant? It is one of the primary humectants in current e-cigarettes so I’m not sure where this is coming from. Also, it is said in the discussion that nicotine is a toxicant but I think there are people who would disagree with this.

**RESPONSE 9:** Propylene glycol is non-toxic when delivered orally. However, inhaling propylene glycol from e-cigarette vapors could have cytotoxic effects on lung epithelial cells. We have revised the sentence as “…*several chemicals in e-cigarette aerosols are known pulmonary toxicants when inhaled (e.g., propylene glycol and diacetyl).*”

We have also modified the sentence in Discussion section: “…*as higher levels of nicotine and toxicant (e.g., tobacco-specific nitrosamines, volatile organic compounds, and metals) have been detected among dual users than among cigarette-only smokers.*”

**COMMENT 10:** The present analyses covered a wide age range, going from 18 years to over 65 years. However, e-cigarette use is uncommon among older adults. Did the authors do sensitivity analyses testing for interactions with age? If not, why not.

**RESPONSE 10:** We performed the analyses among young adults aged 18 – 34 years and among adults aged 18 – 64 years, respectively. Results are similar to those observed in the entire adult sample aged ≥ 18 years. Because findings are universal across age groups, we decided to report results for the entire sample rather than by age group.

**For adults aged 18 – 34 years:**

- Among never combustible tobacco smokers, e-cigarette use was not associated with increased odds of important respiratory symptoms at Wave 5 (former e-cigarette use: AOR = 1.91, 95% confidence interval (CI): 0.82 –4.50, *p* = .136; current e-cigarette use: AOR = 0.63, 95% CI: 0.19 – 2.15, *p* = .457).
- Among former combustible tobacco smokers, e-cigarette use was not associated with important respiratory symptoms (former e-cigarette use: AOR = 1.44, 95% CI: 0.80 – 2.60, *p* = .219; current e-cigarette use: AOR = 0.99, 95% CI: 0.36 – 2.75, *p* = .984).
- Among current combustible tobacco smokers, e-cigarette use was associated with important respiratory symptoms (former e-cigarette use: AOR = 1.89, 95% CI: 1.01 – 3.56, *p* = .048; current e-cigarette use: AOR = 2.05, 95% CI: 1.08 – 3.90, *p* = .030).

**For adults aged 18 – 64 years:**

- Among never combustible tobacco smokers, e-cigarette use was not associated with increased odds of important respiratory symptoms at Wave 5 (former e-cigarette use: AOR = 1.76, 95% CI: 0.84 –3.70, *p* = .135; current e-cigarette use: AOR = 0.80, 95% CI: 0.26 – 2.39, *p* = .674).
- Among former combustible tobacco smokers, former e-cigarette use was associated with a higher odd of important respiratory symptoms (AOR = 1.59, 95% CI: 1.07 – 2.37, *p* = .024), but current e-cigarette use was not associated with such outcome (AOR = 1.55, 95% CI: 0.82 – 2.94, *p* = .175).
- Among current combustible tobacco smokers, e-cigarette use was associated with important respiratory symptoms (former e-cigarette use: AOR = 1.34, 95% CI: 1.00 – 1.78, *p* = .047; current e-cigarette use: AOR = 1.53, 95% CI: 1.12 – 2.09, *p* = .008).
