# Supplementary material for: Longitudinal association between e-cigarette use and respiratory symptoms among US adults: Findings from the Population Assessment of Tobacco and Health Study Waves 4–5
Source: PLoS One. 2024 Feb 29;19(2):e0299834. doi: 10.1371/journal.pone.0299834 (PMC10903800; doi:10.1371/journal.pone.0299834)
Supplement: Attachment — Submitted filename: EC Use and Respiratory Health 20240212 Response.docx [file pone.0299834.s003.docx]

**Additional Editor Comments**
**COMMENT:** Please respond to comments raised by reviewer #2 and specifically comments number 4 and 5.

**RESPONSE:** We appreciate the time that the editor and reviewers have taken to review our work. We have revised our paper accordingly. Please see details below.

**Comments of Reviewer #1**

**COMMENT:** The authors have adequately addressed my comments and I appreciate their thoughtful responses. I have no further comments.

**RESPONSE:** We are grateful for the reviewer’s comment.

**Comments of Reviewer #2**

**COMMENT:** 1. The discussion says the present results are consistent with other studies in finding no association of e-cigarette use with respiratory symptoms among never smokers. That is not correct. There are in fact a number of studies that find exactly the opposite. One difference is that the other studies used actual diagnoses of disease (asthma or COPD) as the criterion. The authors say here that they are studying important respiratory symptoms but ignore research that has studied diagnoses of actual respiratory disease, as if the authors’ approach is the only way to do business and asthma and COPD are unimportant. For that it’s worth, I don’t think this is a correct approach. I think that in discussion, readers should be informed about evidence from all relevant studies. The authors may choose to pass over this comment but that is my view.

**RESPONSE:** We have revised the sentences, which now read as “Consistent with a previous longitudinal epidemiological study [14], we detected no association between e-cigarette use and important respiratory symptoms among never combustible tobacco smokers. But, two previous studies analyzing PATH data found that, for never combustible cigarette smokers, e-cigarette use is associated with incident respiratory symptoms among young adults [16] or worsening of respiratory symptoms among adults [15], suggesting that exclusive e-cigarette use may pose respiratory risks.”

We are aware of cross-sectional studies that detected the significant association between e-cigarette use and respiratory diseases (e.g., asthma and COPD), and we cited those studies in the Introduction. As our study analyzed longitudinal epidemiological data (the PATH Study), we focused on discussing the (consistent and inconsistent) findings from prior studies based on PATH data in the Discussion section.

**COMMENT:** 2. Definition of variables: 2a. The definition of current smoker apparently is not current exclusive smoker; this category includes poly users. Unless I missed something, that is not exclusive cigarette smoking because it includes individuals who used multiple tobacco products. This needs to be made very clear. 2b. Similarly for e-cigarette use, is this really e-cigarette use exclusive of smoking? And of any other tobacco products? And what about marijuana? This is a significant issue because in many samples polyuse is the norm, and readers need to know exactly how the categories were defined. I don't know if the authors should be asked to reanalyze their data but I think this issue needs to highlighted in a revision.

**RESPONSE:** As described in the “Key measures” section, current smokers were defined as “past 30-day use of any combustible tobacco products”, and combustible tobacco products include cigarettes, traditional cigars, cigarillos, filtered cigars, pipes, and hookah.

Regarding marijuana use, we have revised the Limitation section as “…There may be other variables (e.g., marijuana use) affecting the outcome. Future research is warranted to replicate the analyses accounting for those variables (e.g., cigarette pack years and marijuana use [15]).”

**COMMENT:** 3. The presentation of analyses and results is still confusing. When the authors say an analysis is on “important respiratory symptoms,” apparently this means a binary criterion variable. At other points, respiratory symptoms means a continuous index of symptoms. The Ms goes back and forth without justifying why one of these is important and the other isn’t. Apparently, they think this is intuitively obvious but it isn't obvious to me.

**RESPONSE:** We apologize for the confusion. We have revised the “Key measures” section that “When used as the outcome variable, it was coded as a binary variable and an index score of ≥2 was defined as having important respiratory symptoms [17].”

As described in the “Statistical analysis” section, we examined the association between Wave 4 e-cigarette use status (current/former use vs. never use) and Wave 5 respiratory symptom index (≥2 vs. <2) by combustible tobacco smoking status, “adjusting for Wave 4 respiratory symptom index (as a continuous variable)” and other covariables.

In sum, as an outcome variable (Wave 5), the respiratory symptom index was coded as a binary variable. We controlled for Wave 4 respiratory symptom index (covariable) because baseline respiratory symptom index would affect follow-up respiratory symptom index, and as a covariable we used the original value (a continuous variable).

**COMMENT:** 4. It is still unclear to me how the authors can say that their longitudinal analyses are based on participants without any respiratory disease at baseline, and then report the mean number of respiratory symptoms they had at baseline Maybe I’m dense (I’ve been accused of worse) but it’s difficult for me to understand how you can have it both ways.

**RESPONSE:** We have revised the sentences to clarify that respondents were those who “reported no diagnosis of any respiratory diseases”. It is likely that respondents had respiratory symptoms but had never been diagnosed with any respiratory disease. We controlled for baseline respiratory symptom index because baseline respiratory symptoms would affect follow-up respiratory symptoms, that is people with more respiratory symptoms at baseline were more likely to report functionally important respiratory symptoms at follow-up.

**COMMENT:** 5. The authors say that coughing is a measure of chronic airway obstruction as in asthma. I believe that coughing can also be attributable to infectious causes, i.e., upper respiratory infections such as colds or flu.

**RESPONSE:** We completely agree with the reviewer and therefore, have deleted the sentence in the Introduction section.
